# Comprehensive Analysis of Endoplasmic Reticulum Stress in Intracranial Aneurysm

**DOI:** 10.3389/fncel.2022.865005

**Published:** 2022-04-06

**Authors:** Bo Chen, Hongshu Zhou, Xiaoxi Zhou, Liting Yang, Yuanyuan Xiong, Liyang Zhang

**Affiliations:** ^1^Department of Neurosurgery, Xiangya Hospital, Central South University, Changsha, China; ^2^National Clinical Research Center for Geriatric Disorders, Xiangya Hospital, Central South University, Changsha, China; ^3^Department of Neurosurgery, The Second Affiliated Hospital of Nanchang University, Nanchang, China

**Keywords:** intracranial aneurysm, endoplasmic reticulum stress, bioinformatics, drug prediction, unfolded protein response

## Abstract

**Background:**

Aberrant endoplasmic reticulum stress (ERS) plays an important role in multiple cardiovascular diseases. However, their implication in intracranial aneurysms (IAs) remains unclear. We designed this study to explore the general expression pattern and potential functions of ERS in IAs.

**Methods:**

Five Gene Expression Omnibus (GEO) microarray datasets were used as the training cohorts, and 3 GEO RNA sequencing (RNA-seq) datasets were used as the validating cohorts. Differentially expressed genes (DEGs), functional enrichment, Lasso regression, logistic regression, ROC analysis, immune cell profiling, vascular smooth muscle cell (VSMC) phenotyping, weighted gene coexpression network analysis (WGCNA), and protein-protein interaction (PPI) analysis were applied to investigate the role of ERS in IA. Finally, we predicted the upstream transcription factor (TF)/miRNA and potential drugs targeting ERS.

**Results:**

Significant DEGs were majorly associated with ERS, autophagy, and metabolism. Eight-gene ERS signature and IRE1 pathway were identified during the IA formation. WGCNA showed that ERS was highly associated with a VSMC synthesis phenotype. Next, ERS-VSMC-metabolism-autophagy PPI and ERS-TF-miRNA networks were constructed. Finally, we predicted 9 potential drugs targeting ERS in IAs.

**Conclusion:**

ERS is involved in IA formation. Upstream and downstream regulatory networks for ERS were identified in IAs. Novel potential drugs targeting ERS were also proposed, which may delay IA formation and progress.

## Introduction

Intracranial aneurysm (IA) is a life-threatening, complicated, and multifactorial disease that forms owing to the interaction among hemodynamics, genetics, and environmental factors. Immune/inflammation infiltration, cell death, lipid metabolism, oxidative stress, proteolytic activity, and iron accumulation are major histopathological features of IAs (Frösen et al., 2012). The recruitment and infiltration of immune cells have been confirmed to be a key phase in IA formation and development (Hosaka and Hoh, 2014; Signorelli et al., 2018). Recent studies suggest that vascular smooth muscle cell (VSMC) phenotype transformation is crucial to vascular wall remodeling of IA (Starke et al., 2014). Dysregulated autophagy can alter the VSMC phenotype, impair arterial wall function, and contribute to IA formation. Metabolism is also closely associated with the degeneration of IA arterial wall (Frösen et al., 2013). Therefore, it is urgent to investigate the complete mechanisms behind IA formation.

Endoplasmic reticulum stress (ERS) is various physiological or molecular disturbances that unbalance the unfolded-protein-response-regulated endoplasmic reticulum homeostasis (Ren et al., 2021). As a fundamental organelle, the dysfunction of the endoplasmic reticulum can affect multiple biological processes. Relevant studies show that ERS participates in the formation and development of cardiovascular diseases (Ren et al., 2021). Increased ERS markers have been reported in aortic aneurysm walls (Clément et al., 2019). Furthermore, stress-induced ERS can promote VSMC apoptosis, endothelial dysfunction, inflammation infiltration, and ultimately induce aortic aneurysm formation (Jia et al., 2015, 2017). Identifying the associations between ERS and IA may provide a better understanding of IA etiology.

In our study, 5 Gene Expression Omnibus (GEO) microarray datasets were selected as training cohorts, while 3 GEO RNA sequencing (RNA-seq) datasets were selected as validating cohorts. The association between ERS and IA formation was first confirmed by functional enrichment of differential expression genes (DEGs). Afterward, we constructed an ERS signature gene set, identified classical ERS pathways, generated an ERS-VSMC-metabolism-autophagy regulated network, predicted upstream transcription factor (TF) and microRNA targets of ERS genes, and explored the relationship between ERS and single nucleotide polymorphisms (SNPs) in IA diseases. Finally, potential drugs targeting ERS were predicted to inhibit IA formation and development.

## Materials and Methods

### Intracranial Aneurysm Datasets and Preprocessing

Eight public IA datasets were downloaded from the GEO^1^, including 5 microarray datasets (GSE75436, GSE54083, GSE26969, GSE13353, GSE15629) and 3 RNA-sequencing datasets (GSE158558, GSE122897 and GSE66240). The patients involved in the database have obtained ethical approval. The raw data were merged and normalized using the “limma” R package (Ritchie et al., 2015). Batch effects were eliminated using the Combat algorithm (Leek et al., 2012). Of the 181 samples enrolled in our study, microarray data (55IAs and 42 controls) were used as the training set, and RNA-seq data (53 IAs and 31 controls) were used as the validating sets.

### Differentially Expressed Gene Screening and Functional Analysis

Principal components analysis (PCA) was employed to visualize the disparity between IA and control groups using the “factoextra” R package. DEG screening was conducted using the “limma” package (*P* < 0.05 and log2-fold change > 1 or < −1). Furthermore, we analyzed DEG functions by Gene Ontology (GO), Kyoto Encyclopedia of Genes and Genomes (KEGG), and Gene Set Enrichment Analysis (GSEA) analysis (*P* < 0.05).

### Constructing Endoplasmic Reticulum Stress Signature

Two ERS-related gene sets (GO RESPONSE TO ENDOPLASMIC RETICULUM STRESS and GO REGULATION OF RESPONSE TO ENDOPLASMIC RETICULUM STRESS) were downloaded from Molecular Signature Database (MSigDB) v7.0. The Lasso regression was performed to identify the ERS-related DEGs with the highest IA predictive values. The predictive ability was further evaluated by univariate logistic analysis. Next, using these genes, we quantified ERS expression levels of all samples by Gene Set Variation Analysis (GSVA) scores (Hänzelmann et al., 2013).

### Identifying Endoplasmic Reticulum Stress Pathways

Three ERS-related signaling pathways (GOBP ATF6 MEDIATED UNFOLDED PROTEIN RESPONSE, GOBP IRE1 MEDIATED UNFOLDED PROTEIN RESPONSE, GOBP PERK MEDIATED UNFOLDED PROTEIN RESPONSE) were downloaded from MsigDB v7.0. The GSVA scores were performed to quantify the expression level of these pathways in all samples. Pearson correlation analysis was performed between ERS pathways and signature genes.

### Immunocyte Infiltration and Vascular Smooth Muscle Cell Phenotype Analysis

Immunocyte infiltration of arterial walls was estimated using the “xCell” R package, which uses gene expression profiles to predict enrichment of 64 immune and stromal cell types (Aran et al., 2017). The VSMC phenotype was identified by 7 feature genes [SDC1, RBP1, MMP14, CDH2, MGP, PDGFA, MYH9 (Nakahara et al., 1992; Shanahan et al., 1993; Orlandi et al., 2002; Lyon et al., 2010; Chaterji et al., 2014; Shao et al., 2020)] and quantified by GSVA scores.

### Coexpression Analysis of Endoplasmic Reticulum Stress, Intracranial Aneurysm, Immune, and Vascular Smooth Muscle Cell Phenotype

Weighted gene coexpression network analysis (WGCNA) was performed using the “WGCNA” R package (Langfelder and Horvath, 2008). An optimal soft threshold β was set to attain a scale-free topology network. Next, we evaluated the correlation between “ERS” and other pathophysiological traits. “ERS,” “VSMC synthesis,” and “IA” traits had the same high-associated modules (*P* < 0.001 and r > 0.45), which were assumed to be the key modules involved in IA formation and progression. The gene function of key modules was analyzed using GO and KEGG enrichment.

### Constructing Endoplasmic Reticulum Stress-Vascular Smooth Muscle Cell-Metabolism-Autophagy Protein-Protein Interaction and Endoplasmic Reticulum Stress-Transcription Factor-miRNA Networks

Apart from ERS, the DEG functions also included metabolism and autophagy. To evaluate the association between ERS, metabolism, and autophagy, we downloaded 948 metabolism-related genes from the KEGG database^2^, and 232 autophagy-related genes from the HADb database^3^. GSVA scoring and Pearson correlation analysis were then performed. After identifying the correlation, the aforementioned genes, together with high ERS-VSMC-IA-associated module genes were then imported into the STRING database^4^. Protein-protein interaction (PPI) networks were further visualized by Cytoscape software (version 3.9.0). Furthermore, NetworkAnalyst^5^ (Zhou et al., 2019), a comprehensive network visual analytics platform for gene expression analysis, was applied to predict upstream TFs and miRNAs of ERS. Finally, based on ERS signature genes, we constructed ERS-TF-miRNA networks.

### Exploring the Relationship Between Endoplasmic Reticulum Stress and Non-coding Single Nucleotide Polymorphisms

The 80 TFs and 142 nearby genes of regulatory regions which overlapped with IA-associated SNPs, were downloaded from Laarman’s study (Laarman et al., 2018). The integration analysis was used between TFs of the ERS signature and TFs of regulatory regions. The correlation analysis was performed between the ERS signature and genes in proximity to regulatory regions.

### Small Molecular Drug Analysis for Endoplasmic Reticulum Stress Signature Genes

The Connectivity Map (CMAP) website^6^ was applied to explore small molecule drugs with the potential to inhibit IA formation and development. The drugs with negative Raw_cs and high fdr_q_nlog10 values were considered as potential therapeutic agents because they could suppress the expression of ERS signature genes.

### Statistical Analysis

All statistical analyses were conducted using the R software (version 4.0.2). The Wilcox test was applied to compare the difference of continuous variables between the two groups. *P* < 0.05 was considered statistically significant. Data were visualized using the R package “ggplot2.” Heatmaps were drawn using the “pheatmap” R package. Volcano plots were generated using the “ggrepel” R package.

## Results

### Data Preprocessing and Differentially Expressed Gene Screening

**FIGURE 1:**
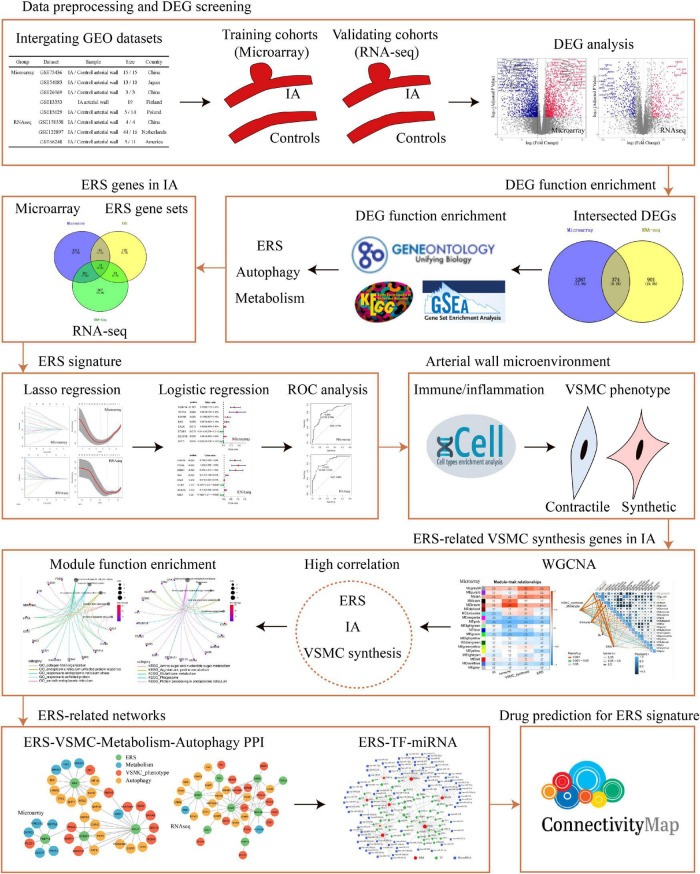
The flow chart of data analysis. DEG, differential expression gene; RNA-seq, RNA sequencing; ERS, endoplasmic reticulum stress; ROC, receiver operating characteristic; VSMC, vascular smooth muscle cell; PPI, protein–protein Interaction; TF, transcription factor.

**FIGURE 2:**
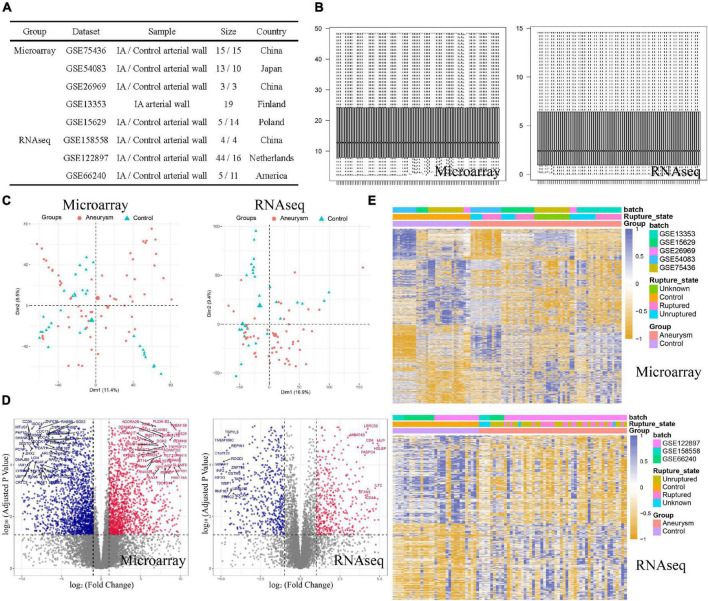
Data preprocessing and DEG screening in both training and validating cohorts. **(A)** Basic information of included dataset. **(B)** Boxplots of normalized data showed similar distributions of different samples. **(C)** PCA plots showed the IA group could be discriminated from the controls. **(D)** Volcano plots visualized the fold change and *P*-value of all genes between the two groups. Red plots were upregulated genes and blue plots were downregulated genes. **(E)** Heatmaps visualized the expression level of DEGs.

The study was designed as indicated in the flow chart (Figure 1). We sought to explore the role of ERS in IA formation by comprehensive analysis based on microarray and RNA-sequencing datasets. In total, we collected 55 cases of IA and 42 cases of normal arteries as controls in microarray training cohorts, and 53 cases of IA and 31 cases of normal arteries as controls in RNA-seq validation cohorts (Figure 2A). For both training and validation cohorts, similar distributions of different samples were observed in normalized data after preprocessing (Figure 2B). PCA analysis showed that the IA group could be discriminated from the controls at the transcript level (Figure 2C). On filtering with the limma package, 1,628 up-regulated genes and 2,013 down-regulated genes were found in the training cohort. 590 up-regulated genes and 685 down-regulated genes were found in the validation cohort (Figure 2D). Heatmaps were used to visualize the expression of DEGs in all cases (Figure 2E).

### Differentially Expressed Gene Functional Enrichment

**FIGURE 3:**
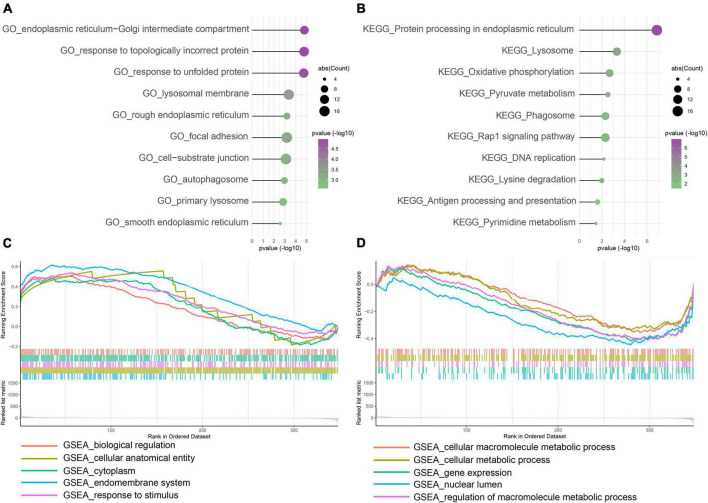
Function enrichment of intersected DEGs between the training and control group. **(A)** Gene Ontology (GO) enrichment analysis. **(B)** Kyoto Encyclopedia of Genes and Genomes (KEGG) enrichment analysis. **(C,D)** Gene Set Enrichment Analysis (GSEA). Enrichment showed that DEG function mainly focused on ERS, unfolded protein response (UPR), autophagy, immune/inflammation, and metabolism.

To explore disease progression in IA, we performed functional enrichment analysis for intersected DEGs between the training and validation cohorts. Among GO enrichment terms, the most overrepresented were ERS, response to unfolded protein, autophagosome, and similar pathways (Figure 3A). In the KEGG pathway analysis, DEGs were notably enriched in protein processing in ER, metabolism process, phagosome, antigen processing and presentation, and others (Figure 3B). In GSEA biological process results, endomembrane system and response to stimulus terms showed higher expression in the IA group (Figure 3C), whereas cellular macromolecule metabolic process was more frequent in the normal artery control group (Figure 3D). Overall, DEGs were functionally enriched in ERS, autophagy, and metabolism-related processes. Further correlation analysis showed that the expression of autophagy and metabolism was positively associated with ERS, separately (Supplementary Figures 1A,B).

### Constructing the Endoplasmic Reticulum Stress Related Signature in Intracranial Aneurysm

**FIGURE 4:**
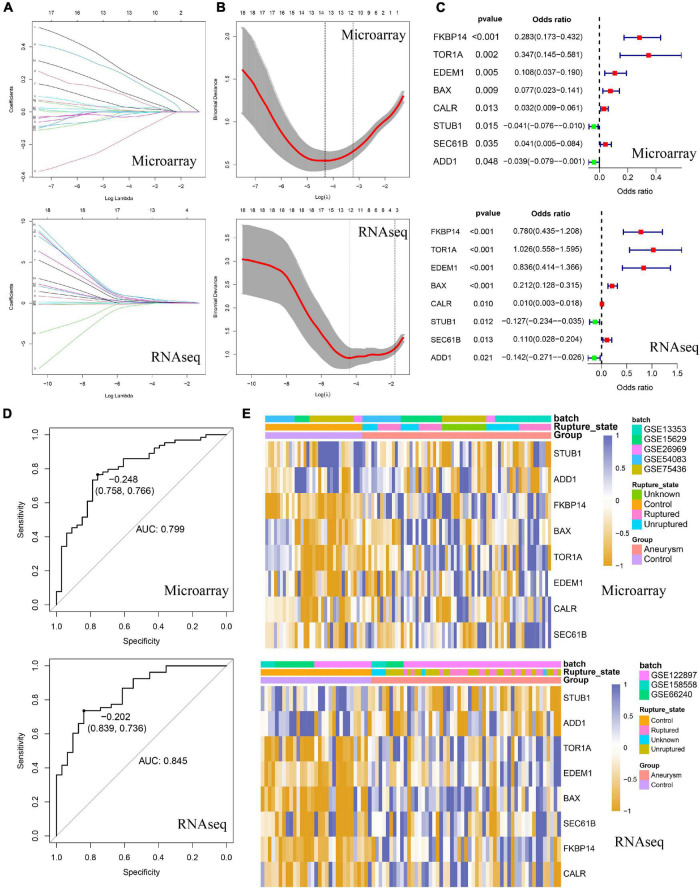
Identification of the ERS-related signature in both training and validating cohorts. **(A)** The coefficient profiles of the LASSO regression model. **(B)** Cross-validation for tuning parameter screening in the LASSO regression model. **(C)** Univariate Logistic regression identified 8 ERS genes’ odds ratios (ORs) and 95% confidence intervals (Cls) after LASSO regression filtration. **(D)** ROC curve analysis for Gene Set Variation Analysis (GSVA) scores of 8 ERS genes. **(E)** The heatmap visualized the expression level of 8 ERS genes.

Considering the prominent role of ERS in DEG functional enrichment, we sought to determine diagnostic values of ERS in IA by constructing an ERS signature. Firstly, we selected 18 IA-related ERS genes by intersecting ERS gene sets and DEGs of training and validation cohorts. Next, these 18 genes were used for LASSO regression to select the most valuable predictive genes, and an 8-gene ERS signature was constructed (Figures 4A,B). FKBP14, TOR1A, EDEM1, BAX, CALR, SEC61B were upregulated, whereas STUB1 and ADD1 were downregulated in IAs (Supplementary Figure 3A). Univariate Logistic regression showed FKBP14, TOR1A, EDEM1, BAX, CALR, and SEC61B may promote IA formation, while STUB1 and ADD1 can prevent it (Figure 4C). ROC curve analysis showed that GSVA scores of the ERS signature could predict IA formation, with areas under the curve (AUC) of 0.799 and 0.845 in the training and validation cohorts, respectively (Figure 4D). Heatmaps were used to visualize ERS signature expression in all cases (Figure 4E).

### Identifying Signaling Pathways in Intracranial Aneurysm

**FIGURE 5:**
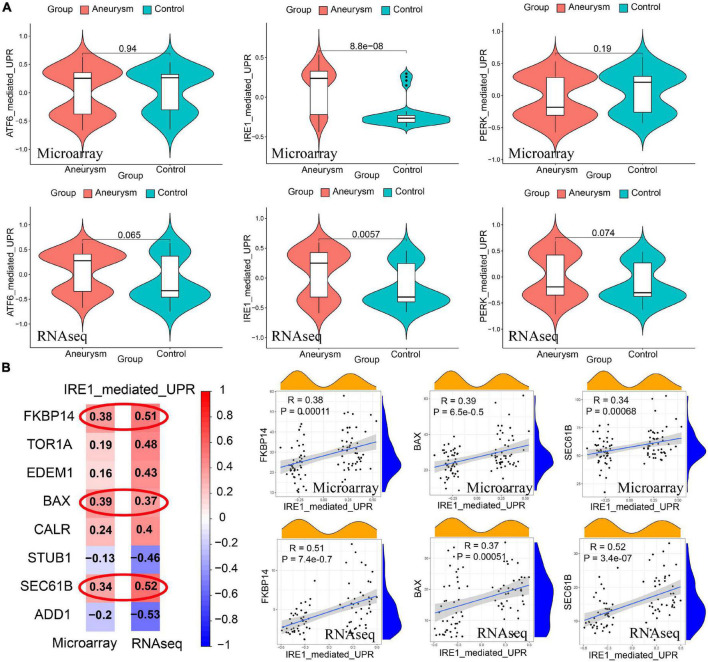
Identification of ERS-related signaling pathways in both training and validating cohorts. **(A)** GSVA scores of 3 pathway expression. IRE1 pathway had a higher expression level in IAs than the level in controls. The expression of the ATF6 pathway and PERK pathway did not show significant differences between the two groups. **(B)** Pearson correlation between IRE1 pathway and signature genes. The expression of FKBP14, BAX, and SEC61B had high correlations to IRE1 pathway expression (Correlation coefficient > 0.3 in both training and validating cohorts).

There were 3 ERS-related classical signaling pathways, including the ATF6 pathway, IRE1 pathway, and Perk pathway. The expression of the IRE1 pathway was significantly higher in IAs than controls, whereas the ATF6 pathway and perk pathway did not show significant differences between the two groups (Figure 5A). IRE1 pathway showed high correlations to FKBP14, BAX, and SEC61B expression (Correlation coefficient > 0.3, Figure 5B).

### Annotation of the Arterial Wall Microenvironment of Immune/Inflammation Infiltrating and Vascular Smooth Muscle Cell Phenotype

**FIGURE 6:**
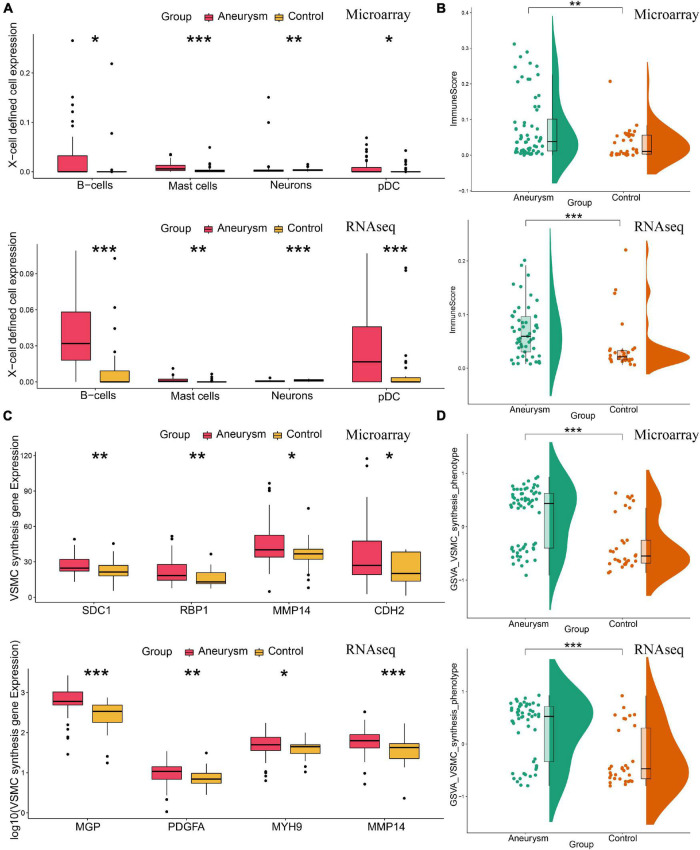
Annotation of the arterial wall microenvironment of immune/inflammation infiltrating and VSMC phenotype in both training and validating cohorts. **(A)** The expression level of X cell-defined immunocytes and neurons. **(B)** The total enrichment scores of the immune microenvironment. IA lesions had higher immune expression than control arteries. **(C)** The expression of feature genes of VSMC synthesis phenotype. **(D)** The GSVA scores of VSMC synthesis phenotype. IA lesions had more VSMC synthesis phenotype than control arteries. *P < 0.05; **P < 0.01; ***P < 0.001.

Since immune infiltration/inflammation and VSMC phenotype are tightly associated with IA formation and progression, we further investigated the arterial wall microenvironment. For both training and validation cohorts, Xcell immune profiling results showed more immune/inflammation-related cell types and higher immune scores in IA (Figures 6A,B). VSMC phenotype analysis revealed that IA cohorts expressed more VSMC-synthesis-phenotype-feature genes and higher synthesis-phenotype GSVA scores (Figures 6C,D).

### Coexpression Analysis Identifying Endoplasmic Reticulum Stress-Related Vascular Smooth Muscle Cell Phenotype Genes

**FIGURE 7:**
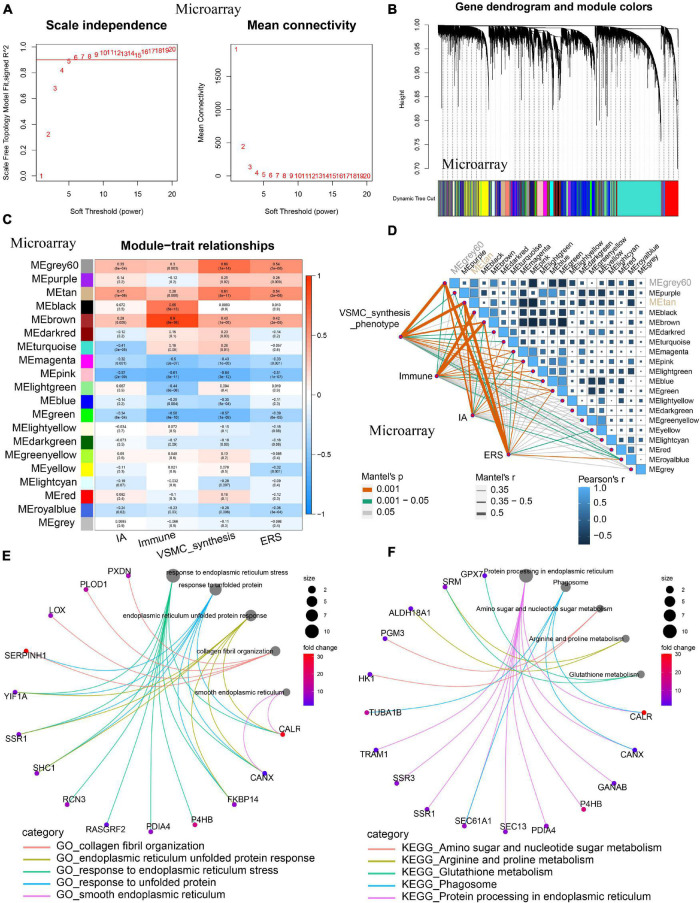
Co-expression analysis identifying ERS-related VSMC phenotype genes in the training cohort. **(A)** A scale-free network construction (power threshold β = 5). **(B)** Gene dendrogram generating gene modules. **(C)** and **(D)** Correlation analysis between modules and pathophysiological traits. IA occurrence, VSMC synthesis, and ERS had the same two highest correlation modules (MEgray60 and MEtan). **(E)** GO enrichment analysis of the intersection between the training cohort (MEgray60 and MEtan) and the validating cohort (MEbrown and MEpink). **(F)** KEGG enrichment analysis of the intersection between the training cohort (MEgray60 and MEtan) and (MEbrown and MEpink). Function enrichment showed that these modules mainly focused on smooth endoplasmic reticulum, collagen fibril organization, metabolism, and phagosome.

Considering ERS, immune/inflammation, and VSMC phenotype are all involved in IA formation, we sought to conduct WGCNA coexpression analysis to identify the relationship among those. Powers β = 5 or 4 were selected as the software threshold for scale-free network construction in training and validation cohorts, respectively (Figure 7A and Supplementary Figure 2A). In the training cohort, 20 modules were identified, and in the validation cohort, 13 modules were identified by clustering dendrogram (Figure 7B and Supplementary Figure 2B). IA, VSMC synthesis, and ERS had the same highest-correlated modules (MEgray60 and MEtan), indicating strong associations among these traits (Figures 7C,D). A similar result was also observed in the validation cohort (Supplementary Figures 2C,D). By intersecting the two most relevant modules in the training and validation group, we identified 85 ERS-related VSMC phenotype genes involved in IA formation. GO enrichment analysis showed these genes mainly focused on collagen fibril organization, smooth endoplasmic reticulum, and others (Figure 7E). KEGG pathway analysis showed metabolism, phagosome, and protein processing in the endoplasmic reticulum were more enriched among these genes (Figure 7F).

### Constructing Endoplasmic Reticulum Stress-Vascular Smooth Muscle Cell-Metabolism-Autophagy Protein-Protein Interaction and Endoplasmic Reticulum Stress-Transcription Factor-miRNA Networks

**FIGURE 8:**
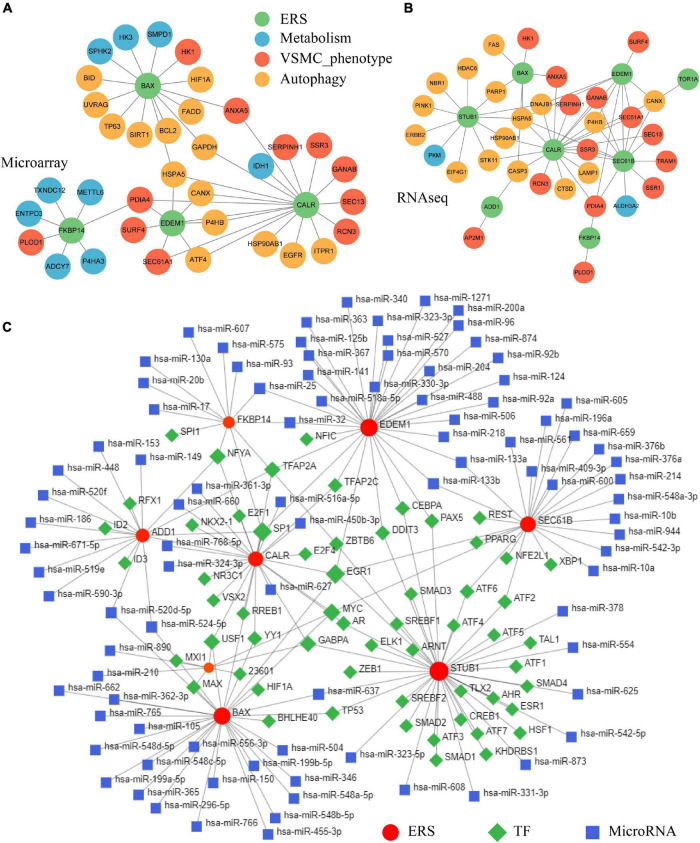
Constructing ERS-VSMC-metabolism-autophagy PPI and ERS-TF-miRNA networks. **(A)** PPI network among ERS, metabolism, VSMC phenotype, and autophagy-related genes in the training cohorts. **(B)** PPI network among ERS, metabolism, VSMC phenotype, and autophagy-related genes in the validating cohorts. **(C)** Prediction of upstream TFs and microRNAs for ERS-related genes.

After identifying the correlation among ERS, VSMC phenotype, metabolism, and autophagy in IA formation, we constructed PPI networks among those pathophysiological traits. Within DEGs targeting the ERS signature, a total of 11 were involved in the VSMC synthesis phenotype, 9 were correlated to metabolism, and 15 were associated with autophagy in the training cohort (Figure 8A). The validating cohort also showed similar ERS-VSMC-metabolism-autophagy PPI networks (Figure 8B).

The NetworkAnalyst online tool was used to predict ERS upstream TF and miRNA. Eight ERS signature genes had identified TFs. NFYA, TFAP2A, SP1, EGR1, MYC, GABPA, and USF1 were common TFs among at least 3 genes. ERS signature genes of EDEM1 and BAX had the most predicted miRNAs including hsa-miR-25, hsa-miR-32, hsa-miR-520d-5p, hsa-miR-524-5p, hsa-miR-637, hsa-miR-133b and hsa-miR-133a (Figure 8C).

### Exploring the Relationship Between Endoplasmic Reticulum Stress and Non-coding Single Nucleotide Polymorphisms

We then investigated TFs and nearby genes of ERS-associated non-coding SNPs in IAs. Seventeen TFs were identified to co-regulate ERS and non-coding SNPs. Among these, MYC had the most ERS target genes and TF binding sites (Supplementary Table 1). Moreover, 6 nearby genes were found to differentially express (Supplementary Figure 3B). Correlation analysis showed the tight connectivity between 8 ERS signature genes and 6 nearby genes, in which KCTD15 had the most significant correlations with ERS (Supplementary Figure 3C).

### Drug Prediction for Endoplasmic Reticulum Stress Signature

**FIGURE 9:**
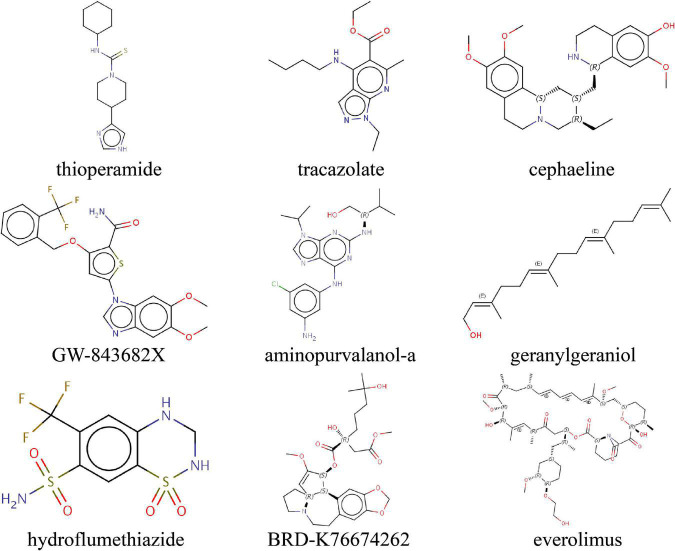
Identifying the molecular structure of 9 small component drugs targeting ERS-related gens in IAs by CMAP.

To predict small molecule drugs with the potential to inhibit IA ERS, we uploaded the ERS signature into the CMAP online tool. We identified 9 drugs (thioperamide, tracazolate, cephaeline, GW-843682X, aminopurvalanol-a, geranylgeraniol, hydroflumethiazide, BRD-K76674262, everolimus) with the negative Raw_cs and the top fdr_q_nlog10 values, suggesting they could inhibit the expression of the ERS signature (Figure 9 and Supplementary Table 2).

## Discussion

Endoplasmic reticulum stress is an imbalance of the endoplasmic reticulum homeostasis caused by an accumulation of unfolded or misfolded proteins. Multiple pathologies can induce ERS, including pressure overload, metabolic disorders, atherosclerosis, ischemia-reperfusion injury, endothelial dysfunction, and others. Long-term ERS promotes abnormal inflammation and apoptosis in the vascular wall, leading to disturbances in cardiovascular function (Ren et al., 2021). Previous studies have shown that excessive ERS is closely associated with various cardiovascular diseases, including heart failure, cardiomyopathy, hypertension, stroke, and the like (Ren et al., 2021).

In the present study, the role of ERS in IA formation was explored using bioinformatics analysis for the first time. We firstly identified ERS by functional enrichment of DEGs, constructed an ERS signature gene set. Afterward, we generated ERS-VSMC-metabolism-autophagy PPI networks and predicted ERS-related upstream TF and microRNAs. The relationship between ERS and non-coding SNPs was then explored. Finally, potential drugs targeting ERS were predicted to inhibit IA formation and development.

Recently, accumulating evidence demonstrates that ERS plays an important role in aneurysm formation and development. In our research, multiple types of DEG functional enrichment analyses showed ERS was related to IA pathogenesis. Similarly, Clément et al. (2019) found increased expression of ERS markers in VSMCs of dissected aortic aneurysms. Jia et al. (2015) proved that stress-induced ERS contributed to thoracic aortic aneurysm and dissection formation. They also reported that ERS-dependent microparticles promote endothelial dysfunction during the formation process of thoracic aortic aneurysm and dissection (Jia et al., 2017). In addition, several studies showed that ERS inhibition could attenuate the formation and development of abdominal aortic aneurysms (Li et al., 2017; Ni et al., 2018).

The ERS signature gene set was then constructed in IA, including FKBP14, TOR1A, EDEM1, BAX, CALR, STUB1, SEC61B, and ADD1. These genes have been confirmed to be related to multiple cardiovascular diseases. Among those, BAX, whose protein belongs to the BCL2 family, is an apoptosis activator. One study showed that overexpressed Bax regulated intimal hyperplasia of VSMCs in arteriosclerosis (Hayakawa et al., 1999). Another study showed that upregulated Bax was associated with the presence of cystic medial degeneration of the aorta (Ihling et al., 1999). Calreticulin (CALR) encoded by the CALR gene, is a highly conserved chaperone protein primarily expressed in the endoplasmic reticulum. Previous studies indicate that CALR can coordinate vascular function and heterocellular calcium signaling (Biwer et al., 2018). STUB1, encoding the protein of STIP1 Homology And U-Box Containing Protein 1, was down-regulated during the IA process in our studies. A prior study showed that the decreased STUB1 in VSMCs inhibited thrombosis in flow loops (Shashar et al., 2017). Adducin 1 (ADD1), belonging to the cytoskeletal protein family, was also expressed at lower levels in vascular walls of IA patients. A sequencing study suggested that ADD1 polymorphism significantly increased the susceptibility to ischemic and hemorrhagic strokes (Kalita et al., 2011).

To update, there are three classic signaling pathways in ERS, including ATF6 pathway, IRE1 pathway, and Perk pathway. They act as proximal sensors of unfolded protein response (UPR) (Wu and Kaufman, 2006). Our results showed that the IRE1 pathway was highly expressed in IA lesions and had strong correlations to the gene expression of BAX, FKBP14, and SEC61B. Previous research has demonstrated that proapoptotic BAX moduled UPR by direct interaction with IRE (Hetz et al., 2006). The overexpression of BAX inhibitor-1 could inhibit IRE and reversed hyperglycemia in diet-induced obesity mice (Bailly-Maitre et al., 2010). Whether these ERS genes could promote IA formation by the IRE1 pathway deserves further basic experimental study.

VSMC phenotype transformation, from contractility to synthesis, is involved in IA formation and development. Our results suggest a strong association between ERS and VSMC synthesis in IA pathogenesis. This relationship has already been confirmed in a previous study. Zhang et al. (2020) showed that the microgravity regulated ERS to induce VSMC phenotype transform. Zhao et al. (2020) identified that Matrine inhibited VSMC phenotype transformation *via* ERS-dependent Notch signaling. Chattopadhyay et al. (2021) found that UPR could drive cholesterol-induced VSMC phenotype transformation.

Considering metabolism and autophagy were also enriched in IAs, we constructed ERS-VSMC-metabolism-autophagy PPI networks. Body metabolism disorders have been discovered to involve the pathological processes of IA. Frösen et al. (2013) found that lipid accumulation and its oxidation in the IA wall, together with low plasma levels of acquired antibodies against oxidized lipids, were associated with IA wall degeneration and rupture. Semmler et al. (2008) demonstrated that polymorphisms of homocysteine metabolism were possible risk factors for IA formation. Besides, growing evidence showed that autophagy was also involved in IA formation, development, and rupture. Sun et al. (2017) proved that ruptured IA tissues had more expression of autophagy-related genes, including LC3, Atg5, and Atg14, followed by unruptured IA and control artery tissues. *In vitro* experiments showed that activated VSMC autophagy could enhance the VSMC proliferation and migration, and induce IA formation (Zhang et al., 2019). Furthermore, the relationships among ERS, metabolism, and autophagy have been demonstrated in other diseases. There are mutual regulations between ERS and metabolism. Fu et al. (2011) showed that aberrant lipid metabolism would cause ERS in obesity. Henkel et al. (2017) proved that ERS regulated hepatic bile acid metabolism in mice. As for autophagy, it is generally considered the last means to restore the homeostasis of the endoplasmic reticulum (Henkel et al., 2017). Together, we speculated ERS can influence metabolism/autophagy/VSMC phenotype and thus contribute to IA formation, which needs further basic research.

The dysregulation of upstream TF and microRNA for ERS also has a crucial impact on the formation and development of cardiovascular diseases. Our TF prediction showed that NFYA, TFAP2A, SP1, EGR1, MYC, GABPA, and USF1 were common TFs with at least 3 ERS genes in IAs. Among these, SP1, whose encoding protein is involved in cell differentiation and growth, has been confirmed to be associated with ERS and VSMC phenotype switching. Dauer et al. (2017) proved that inhibition of SP1 prevented endoplasmic reticulum homeostasis. Hu et al. (2021) found that SP1 regulated migration and phenotype switching of VSMCs through the MAPK pathway in aortic dissections. Tang et al. (2017) identified that microRNA-124 controlled VSMC phenotypic switching *via* SP1. EGR, belonging to the early growth response family, was found to be related to ERS and aneurysm formation. Previous studies showed that ERS can activate EGR1 transcription *via* the MAPK pathway (Shan et al., 2019). Other studies prove that EGR1 upregulation leads to aortic aneurysm formation and EGR1 downregulation can reverse this process (Lin et al., 2020; Shin et al., 2020). In addition, we predicted 91 upstream microRNAs for ERS. Seven microRNAs had 2 target ERS genes. Among these, hsa-miR-25, hsa-miR-133b, and hsa-miR-133a have been confirmed to independently predict aneurysm occurrence or prevent aneurysm development (Li et al., 2014; Plana et al., 2020; Akerman et al., 2021). Furthermore, upregulated hsa-miR-637 can aggravate ERS-induced apoptosis (Kong et al., 2020).

IA-associated SNPs were reported to be enriched in Cow regulatory regions (Laarman et al., 2018). The relationship between non-coding SNPs and ERS was investigated. Integration analysis showed that 17 TFs co-regulated ERS and regulatory regions in IAs. Among these, MYC, whose encoded a nuclear phosphoprotein, with a role in cycle progression, apoptosis, and cellular transformation, had the most ERS target genes and TF binding sites. Previous research had confirmed that MYC was involved in ERS. Dong et al. (2019) found that the IRE1 ERS sensor could activate natural killer cell immunity by MYC regulation. Jayasooriya et al. (2018) found that camptothecin enhanced MYC-mediated ERS and led to autophagy. In addition, Li et al. (2020) proved that the downregulating MYC-mediated ENC1 could prevent IA formation. Correlation analysis showed the tight connectivity between ERS signature genes and nearby genes of regulatory regions. KCTD15, a potassium channel encoding gene, had the most significant associations with ERS. Previous research found that potassium channels have the modification of gating properties under the ERS and were involved in the cerebral vasospasm after subarachnoid hemorrhage (Sobey and Faraci, 1998; Khodaee et al., 2014). The role of KCTD15 in IA formation deserves further research.

Previous studies reported that ERS was the potential therapeutic target for aneurysms. In this research, we predicted 9 small molecule drugs for IAs. These drugs have shown the potential to inhibit ERS progress. Cephaeline and BRD-K76674262, belonging to protein synthesis inhibitors, could inhibit tumor viability, migration, and proliferation (Silva et al., 2021). Han et al. (2013) proved ERS increased protein synthesis leading to cell death, and presented, limiting protein synthesis would be therapeutic for ERS-caused diseases. Everolimus, an mTOR inhibitor, is used in immunosuppressive treatment after organ transplantation and anticancer treatment for advanced renal cell cancers (Patel and Kobashigawa, 2006; Mariniello et al., 2012). Previous studies have found bidirectional crosstalk between ERS and mTOR (Appenzeller-Herzog and Hall, 2012). Persistent mTOR activation could induce ERS occurrence (Wang et al., 2016). Of note, everolimus has been shown capable of limiting aortic aneurysm dilatation in apolipoprotein E-deficient mouse (Moran et al., 2013). IA progress may be delayed by these compounds.

Our study had some limitations. One major limitation was the lack of basic experimental data to confirm and support our findings. Another limitation was the lack of IA-associated clinical data, like size, location, number, and others, to further explore the association between ERS and IA. Additionally, the predicted TF, miRNA, and drugs remain to be further explored to understand their real-world roles in IA formation and development.

## Conclusion

Our results strongly suggest that ERS is involved in IA formation. Upstream and downstream regulatory networks for ERS were identified in IAs. Novel potential drugs targeting ERS were also proposed, which may delay IA formation and progress.

## Data Availability Statement

The original contributions presented in the study are included in the article/Supplementary Material, further inquiries can be directed to the corresponding author/s.

## Ethics Statement

The studies involving human participants were reviewed and approved by open-source GEO database. The patients/participants provided their written informed consent to participate in this study. Written informed consent was obtained from the individual(s) for the publication of any potentially identifiable images or data included in this article.

## Author Contributions

BC, YX, and LZ designed and drafted the manuscript. BC, HZ, LY, XZ, and LZ organized figure legends and revised the article. BC conducted data analysis. All authors have read and approved the final manuscript.

## Conflict of Interest

The authors declare that the research was conducted in the absence of any commercial or financial relationships that could be construed as a potential conflict of interest.

## Publisher’s Note

All claims expressed in this article are solely those of the authors and do not necessarily represent those of their affiliated organizations, or those of the publisher, the editors and the reviewers. Any product that may be evaluated in this article, or claim that may be made by its manufacturer, is not guaranteed or endorsed by the publisher.

## Footnotes

1
https://www.ncbi.nlm.nih.gov/geo/

2
https://www.kegg.jp/

3
http://www.autophagy.lu/

4
https://www.string-db.org/

5
https://www.networkanalyst.ca/

6
https://clue.io/

## Supplementary Material

Supplementary Figure 1 Correlation analysis among ERS, metabolism, and autophagy in both training and validating cohorts. Autophagy **(A)** and metabolism **(B)** were positively associated with ERS, separately.

Supplementary Figure 2 Co-expression analysis identifying ERS-related VSMC phenotype genes in the validating cohort. **(A)** A scale-free network construction (power threshold β = 4). **(B)** Gene dendrogram generating gene modules. **(C,D)** Correlation analysis between modules and pathophysiological traits. IA occurrence, VSMC synthesis, and ERS had the same two highest correlation modules (MEbrown and MEpink).

Supplementary Figure 3 Correlation analysis between ERS and nearby genes of regulatory regions in both training and validating cohorts. **(A)** The expression level of ERS signature genes. IA lesions had higher expression of FKBP14, TOR1A, EDEM1, BAX, CALR, SEC61B, and lower expression of STUB1 and ADD1. **(B)** Venn diagrams of differential expressed genes (DEGs) and nearby genes. Six nearby DEGs of regulatory regions were gained. **(C)** Correlation analysis between ERS signature genes and nearby genes. KCTD15 had the most significant associations with ERS.

Supplementary Table 1 TF intersection between ERS signature genes and regulatory regions.

Supplementary Table 2 Identified 9 small molecular drugs by CMAP.

## Abbreviations

ADD1: adducin 1
AUC: areas under the curve
CALR: calreticulin
CMAP: connectivity map
DEG: differential expression gene
ERS: endoplasmic reticulum stress
GEO: Gene Expression Omnibus
GO: Gene Ontology
GSEA: Gene Set Enrichment Analysis
GSVA: Gene Set Variation Analysis
IA: intracranial aneurysm
KEGG: Kyoto Encyclopedia of Genes and Genomes
PCA: principal components analysis
PPI: protein–protein Interaction
RNA-seq: RNA sequencing
SNP: single nucleotide polymorphism
TF: transcription factor
UPR: unfolded protein response
VSMC: vascular smooth muscle cell
WGCNA: weighted gene coexpression network analysis.
