# Supplementary figures and images for: Comprehensive Analysis of Endoplasmic Reticulum Stress in Intracranial Aneurysm
Source: Front Cell Neurosci. 2022 Apr 6;16:865005. doi: 10.3389/fncel.2022.865005 (PMC9022475; doi:10.3389/fncel.2022.865005)

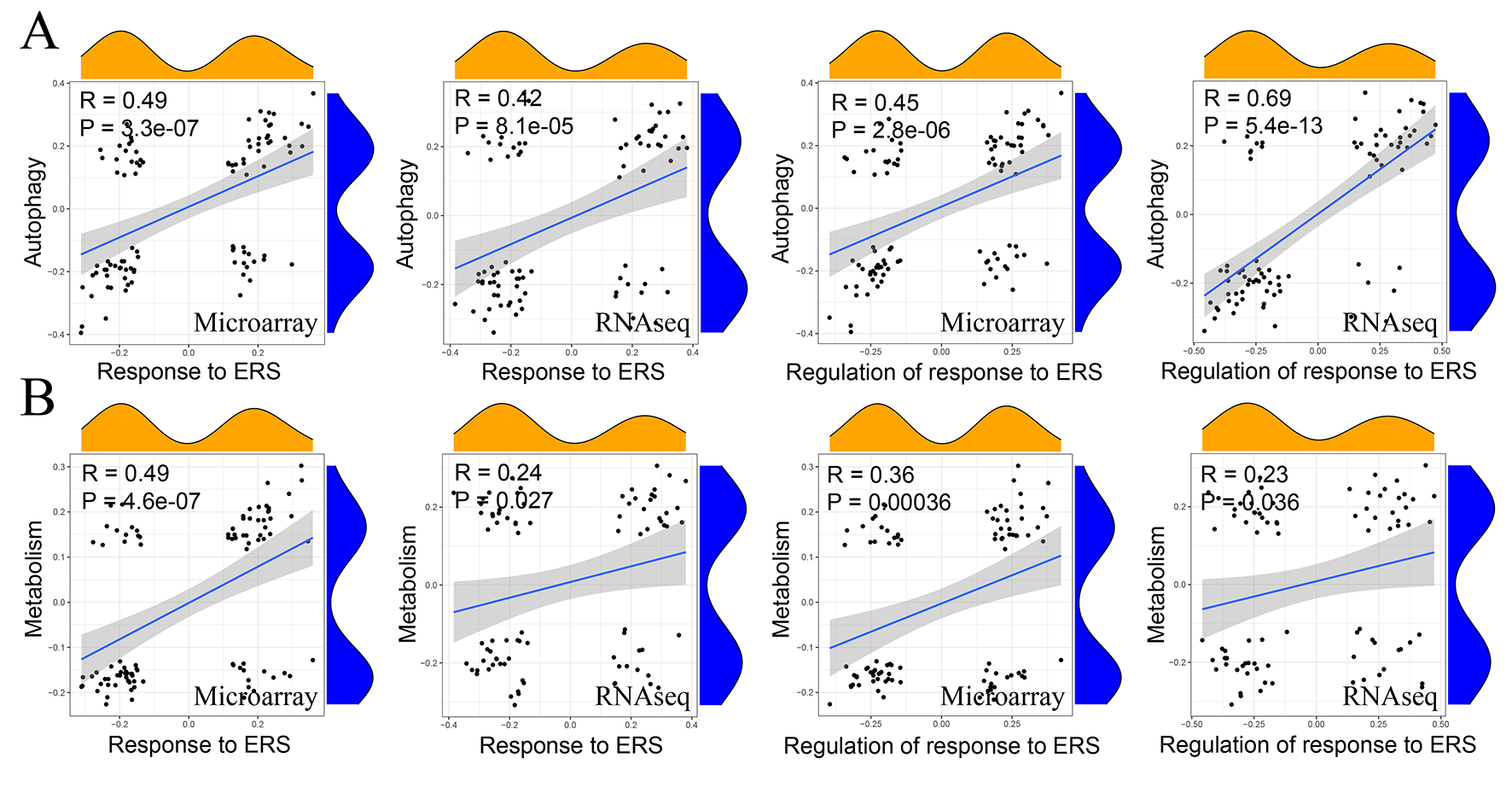

Supplement: Supplementary Figure 1 — Correlation analysis among ERS, metabolism, and autophagy in both training and validating cohorts. Autophagy (A) and metabolism (B) were positively associated with ERS, separately. [file Image_1.TIF]

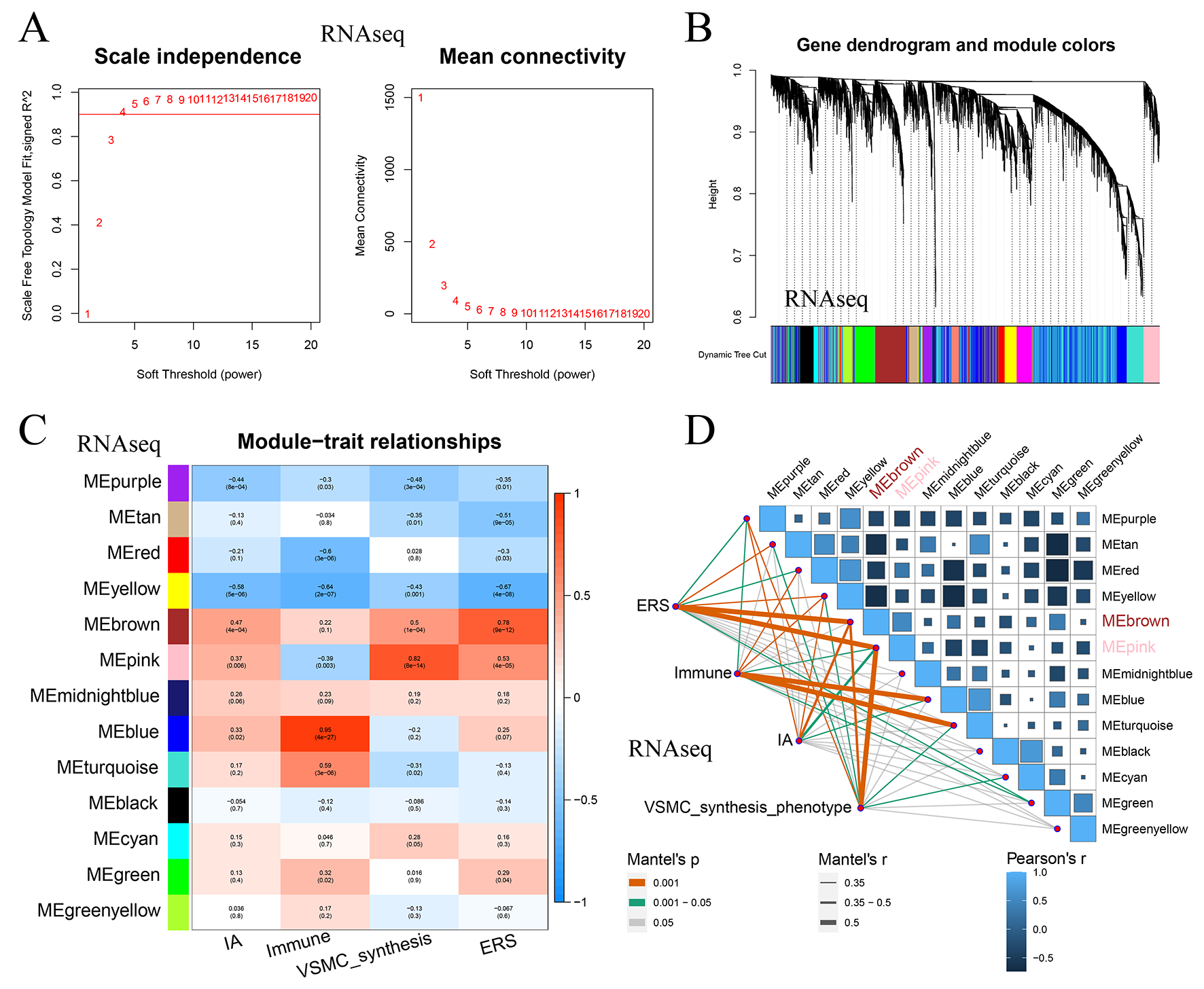

Supplement: Supplementary Figure 2 — Co-expression analysis identifying ERS-related VSMC phenotype genes in the validating cohort. (A) A scale-free network construction (power threshold β = 4). (B) Gene dendrogram generating gene modules. (C,D) Correlation analysis between modules and pathophysiological traits. IA occurrence, VSMC synthesis, and ERS had the same two highest correlation modules (MEbrown and MEpink). [file Image_2.TIF]

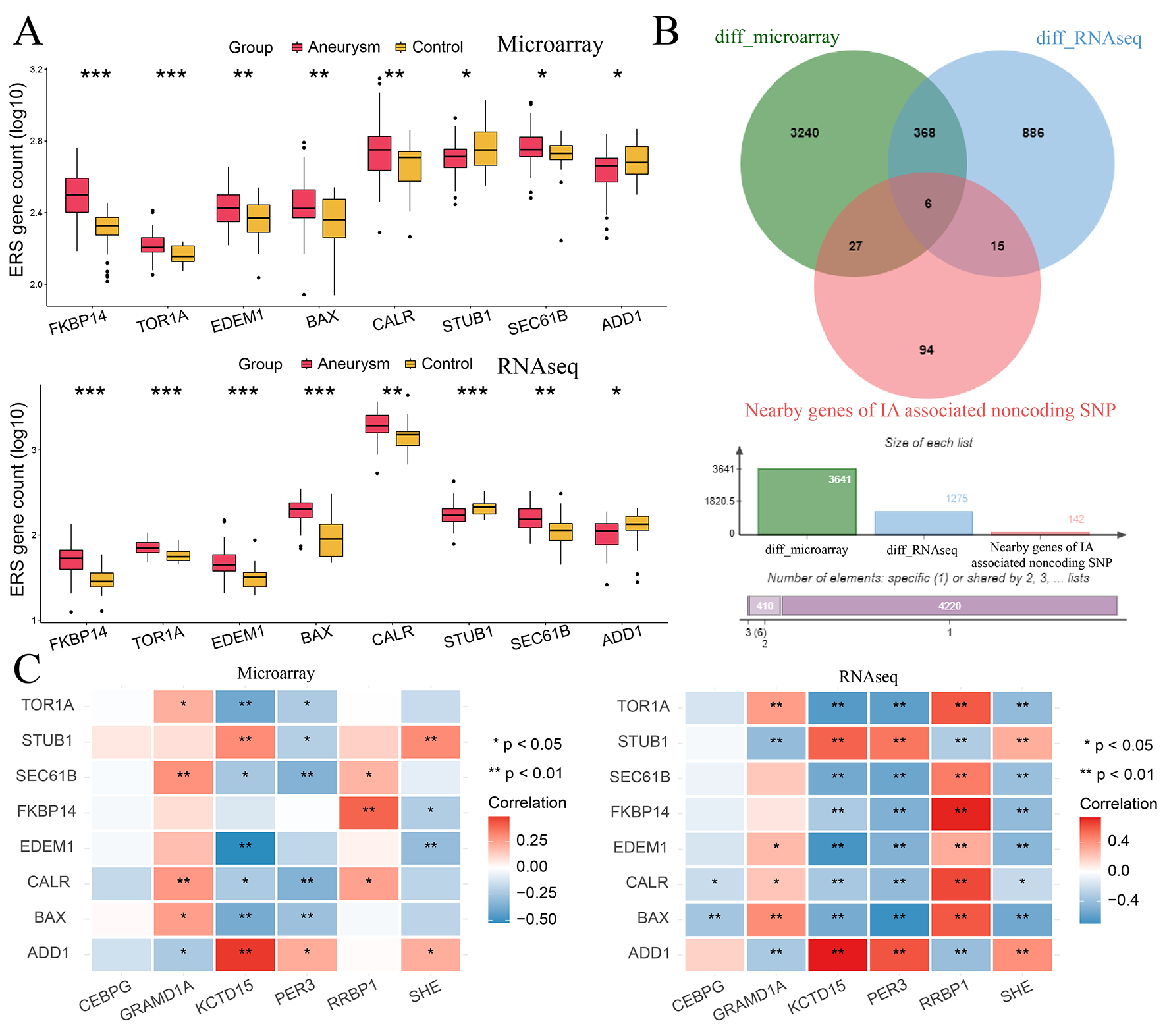

Supplement: Supplementary Figure 3 — Correlation analysis between ERS and nearby genes of regulatory regions in both training and validating cohorts. (A) The expression level of ERS signature genes. IA lesions had higher expression of FKBP14, TOR1A, EDEM1, BAX, CALR, SEC61B, and lower expression of STUB1 and ADD1. (B) Venn diagrams of differential expressed genes (DEGs) and nearby genes. Six nearby DEGs of regulatory regions were gained. (C) Correlation analysis between ERS signature genes and nearby genes. KCTD15 had the most significant associations with ERS. [file Image_3.TIF]
