# Supplementary material for: Comprehensive Analysis of Endoplasmic Reticulum Stress in Intracranial Aneurysm
Source: Front Cell Neurosci. 2022 Apr 6;16:865005. doi: 10.3389/fncel.2022.865005 (PMC9022475; doi:10.3389/fncel.2022.865005)
Supplement: Supplementary Table 1 — TF intersection between ERS signature genes and regulatory regions. [file Table_1.docx]

Supplemental table1 TF intersection between ERS signature genes and regulatory regions

| id | Target ERS gene numbers | Number of TF binding sites in all regions combined | Number of regulatory regions containing this TF binding site |
| --- | --- | --- | --- |
| MYC | 4 | 6 | 3 |
| SP1 | 4 | 1 | 1 |
| USF1 | 3 | 2 | 1 |
| GABPA | 3 | 1 | 1 |
| NFYA | 3 | 1 | 1 |
| MAX | 2 | 6 | 3 |
| ATF2 | 2 | 3 | 2 |
| YY1 | 2 | 3 | 2 |
| TFAP2C | 2 | 1 | 1 |
| MXI1 | 1 | 3 | 2 |
| NFIC | 1 | 2 | 2 |
| BHLHE40 | 1 | 1 | 1 |
| E2F4 | 1 | 1 | 1 |
| ELK1 | 1 | 1 | 1 |
| ESR1 | 1 | 1 | 1 |
| NR3C1 | 1 | 1 | 1 |
| REST | 1 | 1 | 1 |
