# Supplementary material for: Comprehensive Analysis of Endoplasmic Reticulum Stress in Intracranial Aneurysm
Source: Front Cell Neurosci. 2022 Apr 6;16:865005. doi: 10.3389/fncel.2022.865005 (PMC9022475; doi:10.3389/fncel.2022.865005)
Supplement: Supplementary Table 2 — Identified 9 small molecular drugs by CMAP. [file Table_2.docx]

Supplemental table 2 Identified 9 small molecular drugs by CMAP

| Rank | CMAP name | MOA | Raw_cs | fdr_q_nlog10 |
| --- | --- | --- | --- | --- |
| 1 | thioperamide | Histamine receptor antagonist | -0.85 | 15.65 |
| 2 | tracazolate | GABA receptor antagonist | -0.82 | 15.65 |
| 3 | cephaeline | Protein synthesis inhibitor | -0.81 | 15.65 |
| 4 | GW-843682X | PLK inhibitor | -0.81 | 15.65 |
| 5 | aminopurvalanol-a | Tyrosine kinase inhibitor \| CDK inhibitor | -0.80 | 15.65 |
| 6 | geranylgeraniol | Farnesyltransferase inhibitor | -0.80 | 15.65 |
| 7 | hydroflumethiazide | Sodium channel inhibitor | -0.80 | 15.65 |
| 8 | BRD-K76674262 | Protein synthesis inhibitor | -0.79 | 15.65 |
| 9 | everolimus | MTOR inhibitor | -0.78 | 15.65 |
